# Quantifying differences in packaged food and drink purchases among households with diet-related cardiometabolic multi-morbidity: a cross-sectional analysis

**DOI:** 10.1186/s12889-022-14626-3

**Published:** 2022-11-17

**Authors:** Iben M. Ricket, Jeremiah R. Brown, Todd A. MacKenzie, Yu Ma, Dhruv Grewal, Kusum L. Ailawadi, Jennifer A. Emond

**Affiliations:** 1 Department of Epidemiology, Geisel School of Medicine at Dartmouth, Hanover, NH USA; 2 Department of Biomedical Data Science, Geisel School of Medicine at Dartmouth, Lebanon, NH USA; 3 Desautels Faculty of Management, McGill University, Montreal, Quebec Canada; 4 Marketing Division, Babson College, Babson Park, MA USA; 5 Tuck School of Business at Dartmouth, Hanover, NH USA; 6 Department of Pediatrics, Geisel School of Medicine at Dartmouth, Lebanon, NH USA

**Keywords:** Diet-related cardiometabolic multi-morbidity, Packaged food, and drinks, Energy and nutrient-density, Diet and multi-morbidity

## Abstract

**Background:**

Diet is important for chronic disease management, with limited research understanding dietary choices among those with multi-morbidity, the state of having 2 or more chronic conditions.

The objective of this study was to identify associations between packaged food and drink purchases and diet-related cardiometabolic multi-morbidity (DRCMM).

**Methods:**

Cross-sectional associations between packaged food and drink purchases and household DRCMM were investigated using a national sample of U.S. households participating in a research marketing study. DRCMM households were defined as household head(s) self-reporting 2 or more diet-related chronic conditions. Separate multivariable logistic regression models were used to model the associations between household DRCMM status and total servings of, and total calories and nutrients from, packaged food and drinks purchased per month, as well as the nutrient density (protein, carbohydrates, and fat per serving) of packaged food and drinks purchased per month, adjusted for household size.

**Results:**

Among eligible households, 3795 (16.8%) had DRCMM. On average, households with DRCMM versus without purchased 14.8 more servings per capita, per month, from packaged foods and drinks (*p* < 0.001). DRCMM households were 1.01 times more likely to purchase fat and carbohydrates in lieu of protein across all packaged food and drinks (*p* = 0.002, *p* = 0.000, respectively). DRCMM households averaged fewer grams per serving of protein, carbohydrates, and fat per month across all food and drink purchases (all *p* < 0.001). When carbonated soft drinks and juices were excluded, the same associations for grams of protein and carbohydrates per serving per month were seen (both *p* < 0.001) but the association for grams of fat per serving per month attenuated.

**Conclusions:**

DRCMM households purchased greater quantities of packaged food and drinks per capita than non-DRCMM households, which contributed to more fat, carbohydrates, and sodium in the home. However, food and drinks in DRCMM homes on average were lower in nutrient-density. Future studies are needed to understand the motivations for packaged food and drink choices among households with DRCMM to inform interventions targeting the home food environment.

**Supplementary Information:**

The online version contains supplementary material available at 10.1186/s12889-022-14626-3.

## Introduction

Multi-morbidity is a medical condition defined by the co-occurrence of two or more chronic diseases and is associated with early mortality, increased disability, decreased quality of life, and greater healthcare utilization [1–5]. In the United States (U.S.), the prevalence of multi-morbidity is approximately 20%, increasing to 40% when obesity is counted as a chronic condition [6, 7]. However, little is known about disease management for multi-morbidity because most research has focused on single-disease outcomes (e.g., diabetes, heart disease etc.) [1, 8, 9]. Thus, patients with multi-morbidity often receive conflicting or redundant information on managing their chronic conditions [1].

The adoption of a healthy lifestyle is important for prevention and management of multi-morbidity [1, 9]. Central to a healthy lifestyle is dietary behavior [10]. A low-quality dietary pattern is a well-documented contributor to adverse health outcomes, including several chronic conditions [10, 11]. Moreover, a sub-optimal diet is believed to be a leading cause of preventable death and disability [11]. While clinical nutrition and medical care have well-researched dietary and clinical guidelines for single chronic conditions, comparable guidelines are limited for multiple chronic conditions [1, 9]. Thus, it is unclear how multiple chronic conditions may impact diet choices and whether those managing multiple chronic conditions are adhering to health-promoting dietary practices.

Packaged food and drinks are products sold in packing or packaging [12, 13]. These products are typically processed, with added sodium, sugar, and fat and other additives to improve product flavor, appearance, or texture or to increase shelf-stability [12–14]. Packaged food and drinks predominate the U.S. food supply [12], accounting for approximately 75% of total energy consumed in the U.S. [15]. A diet high in processed packaged food and drinks is an emerging risk factor for obesity and several non-communicable diseases [13, 14] including several cancers [16]. Research specifically to understand purchasing patterns of packaged food and drinks among households with multi-morbidity is lacking. Addressing this gap is critical to understand if the home food environment and dietary choices align with a pattern that best supports health for those with multi-morbidity.

The objective of this study was to quantify patterns of packaged food and drink purchases among households with and without diet-related multi-morbidity. Leveraging the availability of packaged food and drinks in the home to reflect the home food environment offers an important methodological advantage as these products have Universal Product Codes (UPCs), which can be linked to nutritional databases to provide standardized nutritional measures [17, 18]. Prior research using packaged food and drink purchases to study diet choices and health demonstrated that availability of sugar and carbohydrates decreased but availability of fat and sodium increased following a diabetes diagnosis in the household, and that homes with young children classified as overweight or obese were marked by a greater per capita availability of fat and sodium in the home [17, 19]. Given the nascency of this research topic, this cross-sectional study provides an early opportunity to explore associations between packaged food and drink purchases among those with and without diet-related multi-morbidity specifically. Results of this study can contribute to the development of interventions targeting the home food environment to best support healthy dietary choices when managing diet-related chronic diseases.

## Methods

### Study overview

This cross-sectional study used 2005–2009 data from a national household panel managed by Information Resources Inc. (IRI, formally Symphony IRI). Households participating in the panel provided data on packaged food and drink purchases, health status, and demographics to IRI. Participating households scanned the UPC (bar code) on all packaged food and drink purchases brought into the home using a hand-held UPC scanner. The nutritional content and package size (e.g., volume) of each purchased item were obtained by linking its UPC (bar code) to a commercially available nutritional database. Participating households also completed a demographics survey and an annual health survey administered from 2005 to 2008. However, only the demographics survey from 2005 was available for this study. Participants provided informed consent to IRI to participate in the study, and earned points that could be redeemed for discounts, gift cards, etc. in return for their participation. This secondary analysis was exempt from IRB approval at our institution (Dartmouth College).

### Packaged food and drink purchases

Data included food and drink purchases from grocery stores, drug stores, warehouse/club stores, and mass retailers. The study focused on purchases from the 13 largest categories of packaged foods and drinks available in the U. S at the time of the study [17], which included: cereal, cheese, cookies, carbonated soft drinks (CSDs), crackers, frozen dinners, ice cream, juices, milk, processed meats, salty snacks, soup, and yogurt. Data on fresh foods (e.g., meats, seafood, produce, etc.) were not included. However, the U.S. food supply is dominated by packaged food and drinks [12, 15], which are largely of lower nutritional quality marked by added sugars, fats, and sodium [12]. Additionally, because some packaged food and drinks are perceived to be more healthy than others based on cognitive biases about foods or nutritional claims on packaging (i.e., a “health halo bias”) [20], we also analyzed purchases for each food or drink category individually and when classified into two groups using the methods from a previously conducted consumer health survey [17]. Specifically, cereal, cheese, crackers, juice, milk, soup, and yogurt were grouped into items perceived as “healthy”, and cookies, CSDs, ice cream, frozen dinners, processed meats, and salty snacks were grouped into items perceived as “unhealthy”.

The exact package size and nutritional content of each product (calories, protein, carbohydrates, fat, fiber, and sodium) was defined by linking the UPCs from each packaged item to a database of nutrient content for individual stockkeeping units (SKUs), purchased from a commercial vendor [17, 18]. Nutrient data were available for 68.5% of SKUs, representing about 90% of purchases tracked by IRI [18]. The nutritional content for items with missing SKUs were imputed from other varieties of the same brand with a non-missing SKU [18]. Serving size information for each item was obtained from a federal database and included the serving size customarily consumed per eating occasion [21].

### Nutritional profile of packaged foods and drinks

Calories were expressed as kilocalories, macronutrients (i.e., protein, carbohydrates, fat) and fiber were expressed in grams, and sodium was expressed in milligrams. Analyses for all nutritional profile metrics were expressed as monthly average weighted “per capita” values, where weights accounted for household size and age composition of the household [22]. The weights were based on expected daily caloric requirements relative to a 2000 cal/day diet for adults and included the following: age 13 years+ were given a weight of 1, age 6–12 years were given a weight of 0.75, and age 2–5 years were given a weight of 0.575 [22].

Three unique nutritional profile metrics were used to characterize monthly packaged food and drink purchases. All three metrics were based on total purchases across the 13 food and drink categories. The first nutritional profile metric was *quantity* and was operationalized as the number of servings per capita, where servings were defined as total volume of an item divided by the size customarily consumed per eating occasion [18]. The second nutritional profile metric represented a combination of quantity and quality (termed *quantity-quality*) and was operationalized as total calories (kcal), protein (g), carbohydrates (g), fat (g), fiber (g), and sodium (mg), all expressed as per capita values [18] (e.g., total grams of protein purchased per capita). The third nutritional profile metric represented *quality* and was operationalized as total calories (kcal), protein (g), carbohydrates (g), fat (g), fiber (g), and sodium (mg) per capita divided by total servings per capita of purchased food and drinks (e.g., protein per capita divided by total servings per capita), to obtain calories, protein, carbohydrates, fat, fiber, and sodium per serving [18].

.

### Diet-related multi-morbid households

The health survey collected information on health status and health behaviors from household members. Household members were asked to indicate how they treated a list of 40 health conditions in the prior 12 months. Specifically, for each health condition respondents were instructed to, “Please indicate how you have treated the health condition below for the past 12 month.” The options included (1) prescription, (2) over the counter, (3) prescription and over the counter or (4) no treatment. If household members did not have the listed health condition, they were instructed to leave the question blank. Household members providing any response (i.e., not leaving the question blank) were considered to have the associated condition. The outcome was defined from responses from household head(s), defined as the person responsible for making most household decisions and could include up to 2 people. Households where the household head(s) self-reported two or more cardiometabolic chronic diseases associated with diet were classified as diet-related cardiometabolic multi-morbid households (DRCMM) [8, 9]. The diet-related chronic conditions were: angina, atherosclerosis, hyperlipidemia, congestive heart failure, diabetes, myocardial infarction, hypertension, or stroke.

### Covariates

Study covariates included age of the head of household, race, annual household income, educational level for the head of household, marital status for head of household, family size, BMI, and physical activity level for the head of household. Where appropriate, the maximum value between the two household heads was used for households with two dual heads. BMI was computed from self-reported height (feet and inches) and weight (lbs.), and physical activity was the self-reported answer from the annual health survey question, “On a weekly basis, how often do you exercise?” The responses included most days, some days, and rarely/never. Information on covariate categorization is available in Additional file 1.

### Sample for analysis

Households with a completed health survey along with packaged food and drink purchases for the 1-year prior to the health survey responses were included in the analysis. Ultimately, this limited the sample to UPC data collected between 2006 and 2008. In addition, households were required to have at least 9 months of purchase data, where total monthly purchases of the 13 food and drink categories were at least $50. In addition, households missing covariate data (defined above) were excluded from analyses. Packaged food and drink purchases and demographic data were at the household level and the health survey was completed by the household head. The unit of analysis was at the household level. Since the outcome and some covariates were measured among household heads, only characteristics of household heads were used in the analysis.

### Statistical analysis

Demographic and socioeconomic characteristics, as well as the 3 unadjusted nutritional profile metrics, were compared across households with and without DRCMM. Unadjusted nutritional profile metrics for the total purchases across all 13 food and drink categories were summarized overall and separately for healthy versus unhealthy foods and drink categories. Chi-Square tests were used for categorical variables and Student’s t-tests for continuous measures. For unadjusted and adjusted analyses, Holm’s step-down procedure was used to adjust for multiple comparisons [23].

Two separate multiple variable analytical methods were used to compare the *quantity-quality* and the *quality* nutritional profile metrics to DRCMM status. For the *quantity-quality* nutritional profile metrics, the nutrient residual analysis method by Willet [24] was utilized to compare differences in the total calories from each energy-bearing nutrient (fat, carbohydrates, and protein) and the total grams of fiber or total milligrams of sodium purchased from all packaged foods and drinks between DRCMM and non-DRCMM households, adjusted for total calories purchased from the packaged food or drinks. Models thus report on how much more or less of nutrient, fiber or sodium the household purchased per capita, per month, compared to the average for all other households, while accounting for total calories purchased [24, 25]. Specifically, linear regression was first used to fit the total calories purchased for each nutrient (or grams of fiber or milligrams of sodium), separately, on the total calories purchased for each household, and the residuals from that model (i.e., the difference between the observed and predicted value from the model per each household) were extracted to use as the independent variables in a second model [24, 25]. Next, a series of 3 logistic regression models were estimated in which each model included 2 energy-bearing nutrients and excluded one, and were further adjusted for fiber, sodium, total calories per capita, and covariates. Thus, the odds ratio for any one energy-bearing nutrient in a model is the association between a 100 cal increase of that nutrient, in exchange for a 100 cal decrease in the nutrient excluded from the model, and DRCMM household status.

For the *quality* nutritional profile metric, standard multivariable logistic regression modeling was used to compare DRCMM status to the average per capita grams of fat, carbohydrates, protein and fiber and milligrams of sodium per serving across all 13 packaged foods and drinks purchased for the home. As such, this model evaluated energy-density of packaged food and drinks available in the home. Models were adjusted for total calories purchased per capita and covariates. Importantly, since the energy-bearing nutrients were expressed per serving (e.g., per capita grams of protein per serving), they do not sum to total calories per capita (i.e., kcal per capita). As such, all energy-bearing nutrients, when expressed per serving, can be included simultaneously in the same model with calories per capita.

Each of the fully adjusted modelling techniques were run for total nutrients across all 13 food and drink categories. The same analyses were repeated separately for perceived healthy and perceived unhealthy food and drinks categories. All analyses were completed using R version 3.6.0. Additional information on variable operationalization is available in Additional file 2.

### Sensitivity analysis

An important methodological difference in the two fully adjusted models should be noted. The first fully adjusted model (using *quantity-quality* nutritional profile metric) controls for calories, both in the generation of the residual and in the model itself. This model evaluates total energy from packaged food and drinks in the household. Whereas the second fully adjusted model (using *quality* nutritional profile metric) controls for servings in the metric (e.g., per capita grams protein/serving) and calories in the model. This model evaluates the nutrient-density (defined as grams nutrient/serving) and the energy-density (defined as calories/serving) of food and drinks in the household. While calories and servings were highly correlated (rho: 0.93, *p* < 0.001), the energy-density of food and drinks affect these models differently. Low energy-dense items, especially drinks, contribute less to the first fully adjusted model, as these items have few nutrients or calories, especially relative to their servings. The servings from these items contribute greatly to the second fully adjusted model. Given these differences, models evaluating energy-density typically remove caloric and non-caloric beverages [26]. To that end, a sensitivity analysis was performed, repeating the second fully adjusted model (using *quality* nutritional profile metric) when CSDs and juices were removed.

## Results

**Fig. 1:**
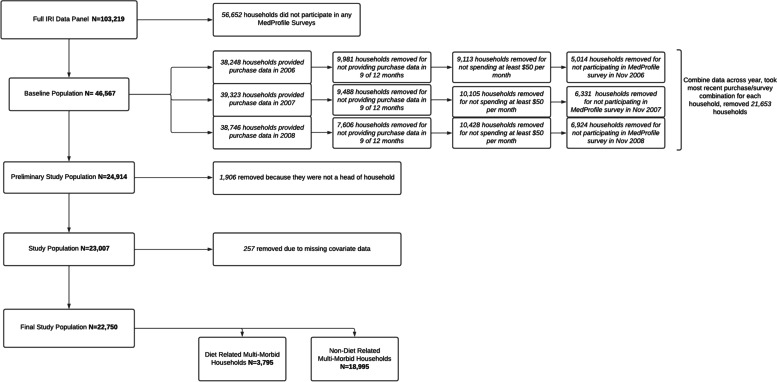
Study Eligibility Flow Chart. This figure illustrates the selection process used to identify eligible households for analysis (*N* = 22,75)

Within the study period (2006–2008), the final eligible study sample included 22,750 households, 3795 (16.7%) of which had DRCMM (Fig. 1). Households with DRCMM were older, had smaller families, were less likely to have children living in the home, and had a lower annual income when compared to household counterparts without DRCMM (Table 1). In addition, household heads from DRCMM households had higher BMI, exercised less frequently, and were more likely to be widowed than households without DRCMM (Table 1).

**Table 1:** Bivariate Statistics, Stratified by Diet-Related Cardiometabolic Multi-Morbidity

	Non-Diet Related Cardiometabolic Multi-Morbid Household	Diet Related Cardiometabolic Multi-Morbid Household^**a**^	*P*-value^**b**^
**N Households**	18,955	3795	
**Survey Year N (%)**			
**2006**	3069 (16.2)	541 (14.3)	0.009
**2007**	5093 (26.9)	1064 (28.0)	
**2008**	10,793 (56.9)	2190 (57.7)	
**Age**	56.36 (12.7)	65.57 (11.6)	< 0.001
**Race N (%)**			
**White**	16,781 (88.5)	3361 (88.6)	< 0.001
**Black**	1009 (5.3)	245 (6.5)	
**Hispanic**	633 (3.3)	116 (3.1)	
**Asian**	281 (1.5)	27 (0.7)	
**Other Race**	251 (1.3)	46 (1.2)	
**Income N (%)**			
**$ 00,000 TO $ 9999**	465 (2.5)	148 (3.9)	< 0.001
**$10,000 TO $11,999**	257 (1.4)	93 (2.5)	
**$12,000 TO $14,999**	335 (1.8)	132 (3.5)	
**$15,000 TO $19,999**	636 (3.4)	210 (5.5)	
**$20,000 TO $24,999**	947 (5.0)	333 (8.8)	
**$25,000 TO $34,999**	1867 (9.8)	530 (14.0)	
**$35,000 TO $44,999**	2178 (11.5)	537 (14.2)	
**$45,000 TO $54,999**	2402 (12.7)	465 (12.3)	
**$55,000 TO $64,999**	1845 (9.7)	333 (8.8)	
**$65,000 TO $74,999**	1806 (9.5)	308 (8.1)	
**$75,000 TO $99,999**	3160 (16.7)	393 (10.4)	
**$100,000 AND GREATER**	3057 (16.1)	313 (8.2)	
**Marital Status N (%)**			
**Single**	1509 (8.0)	259 (6.8)	< 0.001
**Married**	13,808 (72.8)	2627 (69.2)	
**Divorced**	2197 (11.6)	422 (11.1)	
**Widowed**	1229 (6.5)	436 (11.5)	
**Separated**	212 (1.1)	51 (1.3)	
**Family Size N (%)**			
**One Person**	2802 (14.8)	679 (17.9)	< 0.001
**Two People**	7986 (42.1)	2110 (55.6)	
**Three People**	3154 (16.6)	521 (13.7)	
**Four People**	3175 (16.8)	283 (7.5)	
**Five People**	1231 (6.5)	140 (3.7)	
**Six People**	421 (2.2)	37 (1.0)	
**Seven People**	126 (0.7)	11 (0.3)	
**Eight or more People**	60 (0.3)	14 (0.4)	
**Physical Activity N (%)**			
**Most days**	5535 (29.2)	897 (23.6)	< 0.001
**Some days**	7627 (40.2)	1483 (39.1)	
**None/Not at all**	5793 (30.6)	1415 (37.3)	
**BMI mean (SD)**	27.0 (35.7)	28.9 (9.4)	0.204

^a^Diet-related cardiometabolic multi-morbidity defined as households having 2 or more of the following conditions: angina, atherosclerosis, diabetes, hyperlipidemia, hypertension, stroke, myocardial infarction, or congestive heart failure

^b^P-value calculated using Chi square and 2-sample T-test for categorical and continuous variables, respectively

### Unadjusted quantity, quantity-quality & quality

Table 2 provides unadjusted results for *quantity*, *quantity-quality,* and *quality* nutritional metrics for non-DRCMM and DRCMM households. Each metric reflects monthly averages over 1 year, weighted for household size and age composition (e.g., weighed per capita per month). In terms of *quantity*, DRCMM households purchased more total servings per capita per month (*p* < 0.001) across all 13 food and drink categories; however, the difference was greatest for perceived unhealthy food and drink categories, with DRCMM households averaging 12.7 more servings per capita per month from perceived unhealthy foods (p < 0.001) and 2.2 more servings per capita per month from perceived healthy foods (*p* = 0.001) than non-DRCMM households. For *quantity-quality*, DRCMM households purchased more calories and more of all nutrients (protein, carbohydrates, fat, fiber, sodium) per capita per month than non-DRCMM households, and the differences were larger and more significant from perceived unhealthy categories than perceived healthy categories. For example, in perceived unhealthy categories, DRCMM households purchased 22.4 more grams of protein per capita per month (*p* < 0.001) than non-DRCMM households, but in perceived healthy categories, DRCMM households purchased 6.8 more grams per capita per month of protein (*p* = .076). Additionally, DRCMM households averaged more sodium from packaged foods and drinks than non-DRCMM households across both perceived healthy and perceived unhealthy categories (*p* < 0.001, for both). In terms of *quality*, the packaged foods and drinks purchased for the home overall averaged fewer per capita grams of each nutrient per serving per month, except sodium, among DRCMM versus non-DRCMM households. The between group differences were statistically significant for perceived healthy food and drink categories for each nutrient except sodium (all *p* < 0.001). Lastly, DRCMM households purchased more per capita grams of sodium per serving per month from perceived unhealthy categories (*p* < 0.001).

**Table 2:** Unadjusted associations for 3 nutritional profile metrics across all food and drink categories^a^ and grouped into those perceived as healthy^b^ and unhealthy^c^, stratified by diet-related cardiometabolic multi-morbid household status

Outcome Status	*Non-Diet Related Cardiometabolic Multi-Morbid Household mean (SD)*	*Diet Related Cardiometabolic Multi-Morbid Household mean (SD)*	*P-value*^*d*^
**N households**	18,955	3795	
**Quantity Nutritional Profile**^**e**^			
**Servings (#/capita)**	134.0 (72.0)	148.8 (97.7)	< 0.001 ^h^
**Servings Healthy (#/capita)**	63.2 (35.0)	65.4 (45.7)	0.001 ^h^
**Servings Unhealthy (#/capita)**	70.8 (51.4)	83.5 (67.6)	< 0.001 ^h^
**Quantity-Quality Nutritional Profile**^**f**^			
**Protein (g/capita)**	535.4 (277.7)	564.6 (375.9)	< 0.001 ^h^
**Protein Healthy (g/capita)**	337.4 (204.6)	344.2 (259.2)	0.076
**Protein Unhealthy (g/capita)**	198.0 (127.9)	220.4 (168.8)	< 0.001 ^h^
**Carbohydrates (g/capita)**	2270.2 (1346.6)	2375.3 (1664.3)	< 0.001d
**Carbohydrates Healthy(g/capita)**	1165.4 (732.6)	1188.2 (925.1)	0.096
**Carbohydrates Unhealthy (g/capita)**	1104.7 (923.1)	1187.1 (1081.5)	< 0.001 ^h^
**Fat (g/capita)**	615.0 (349.9)	670.6 (473.6)	< 0.001 ^h^
**Fat Healthy (g/capita)**	247.5 (155.3)	256.5 (219.1)	0.003 ^h^
**Fat Unhealthy (g/capita)**	367.5 (248.3)	414.1 (309.5)	< 0.001 ^h^
**Fiber (g/capita)**	79.2 (55.5)	81.7 (62.5)	0.016
**Fiber Healthy (g/capita)**	47.2 (44.9)	48.6 (48.5)	0.092
**Fiber Unhealthy (g/capita)**	32.0 (24.9)	33.1 (27.6)	0.017
**Sodium (mg/capita)**	28,640.1 (1520.0)	32,051.1 (2474.0)	< 0.001 ^h^
**Sodium Healthy (g/capita)**	14,894.7 (8456.1)	16,435.2 (15,376.7)	< 0.001 ^h^
**Sodium Unhealthy (g/capita)**	13,745.5 (9202.5)	15,615.9 (12,303.6)	< 0.001 ^h^
**Calories (kcal/capita)**	16,601.1 (8825.1)	17,623.6 (11,580.4)	< 0.001 ^h^
**Calories Healthy (kcal/capita)**	8211.0 (4625.3)	8412.9 (6270.5)	0.021
**Calories Unhealthy (kcal/capita)**	8390.2 (5823.1)	9210.7 (6952.0)	< 0.001 ^h^
**Quality Nutritional Profiles**^**g**^			
**Protein (g/serving)**	4.1 (1.0)	3.9 (1.1)	< 0.001 ^h^
**Protein Healthy (g/serving)**	2.6 (1.0)	2.4 (1.0)	< 0.001 ^h^
**Protein Unhealthy (g/serving)**	1.5 (0.7)	1.5 (0.7)	0.755
**Carbohydrates (g/serving)**	17.1 (3.9)	16.2 (4.0)	< 0.001 ^h^
**Carbohydrates Healthy(g/serving)**	9.1 (3.8)	8.4 (3.7)	< 0.001 ^h^
**Carbohydrates Unhealthy (g/serving)**	8.1 (3.6)	7.8 (3.7)	< 0.001 ^h^
**Fat (g/serving)**	4.7 (1.3)	4.6 (1.3)	0.002 ^h^
**Fat Healthy (g/serving)**	1.9 (0.8)	1.8 (0.8)	< 0.001 ^h^
**Fat Unhealthy (g/serving)**	2.8 (1.1)	2.8 (1.1)	0.004 ^h^
**Fiber (g/serving)**	0.6 (0.3)	0.6 (0.3)	< 0.001 ^h^
**Fiber Healthy (g/serving)**	0.4 (0.3)	0.4 (0.3)	< 0.001 ^h^
**Fiber Unhealthy (g/serving)**	0.3 (0.1)	0.2 (0.1)	< 0.001 ^h^
**Sodium (mg/serving)**	220.7 (60.0)	118.3 (130.0)	0.071
**Sodium Healthy (mg/serving)**	116.6 (45.3)	117.2 (123.7)	0.615
**Sodium Unhealthy (mg/serving)**	104.1 (44.0)	106.3 (43.7)	0.005 ^h^
**Calories (kCal/serving)**	126.2 (19.6)	121.0 (20.7)	< 0.001 ^h^
**Calories Healthy (kcal/serving)**	63.8 (20.9)	59.1 (20.61)	< 0.001 ^h^
**Calories Unhealthy (kcal/serving)**	62.4 (21.4)	61.9 (21.1)	0.176

*kcal* Kilocalorie, *g* gram, *mg* milligram.

^a^13 food categories include: CSDs, cereals, crackers, ice cream, milk, juices, salty snacks, soup, cheese, cookies, frozen dinners, processed meats, yogurt

^b^ Perceived Healthy = Cereal, cheese, crackers, juices, milk, soup, and yogurt

^c^ Perceived Unhealthy = cookies, CSDs, ice cream, frozen dinners, processed meats, and salty snacks

^d^ *P*-value calculated using Chi square and 2-sample T-test for categorical and continuous variables, respectively

^e^ Expressed as one-year monthly averaged weighed serving per capita

^f^ Expressed as one-year monthly average weighed per capita

^g^ Expressed as one-year monthly average weighed per capita, per serving

^h^ Statistically significant using Holms step-down correction applied for multiple comparison across 13 food and drink categories

### Fully adjusted models using quantity-quality nutritional metric

Table 3 presents results from the fully adjusted logistic regression model evaluating the association of DRCMM status with relative calories from protein, carbohydrates, and fat, each per capita per month, controlling for calories per capita and covariates. Three models are presented using the residual method and evaluate total energy from packaged food and drink purchases. For example, Model 1 demonstrates that DRCMM households were more likely to have greater quantities of fat (OR: 1.01; 95% CI: 1.00, 1.02; *p* = 0.002) and carbohydrates (OR: 1.01; 95% CI: 1.01, 1.02; *p* = 0.000) relative to protein as compared to non-DRCMM households. This can also be seen in models 1b & 1c in which different nutrients were excluded from the model. When stratified by perceived healthy or perceived unhealthy categories, significant effects were observed only for perceived unhealthy categories (Models 1a-3a and 1b-3b), and not for perceived healthy categories. DRCMM households consistently purchased more sodium per capita per month, when considering all 13 packaged foods and drinks overall and when stratified by perceived healthy or perceived unhealthy categories, consistent with the unadjusted analyses.

**Table 3:** Regression coefficients and adjusted^a^ odds ratios of diet-related cardiometabolic multi-morbid households relative to 100 kcal per capita, per month, estimated by various residual models^b^, for purchases from 3 cal sources, fiber, sodium, and calories from all food & drinks^c^ and when grouped into perceived healthy^d^ & unhealthy^e^

		Included	B × 100 kcal	Odds Ratio	Standard Error	95% Confidence Interval	***P*** Value
**Across all 13 Food & Drink Categories**							
	**Excluded**	Fat	0.01	1.01	0.00	1.00, 1.02	0.002^g^
**Model 1**	**Protein**	Carbohydrates	0.01	1.01	0.00	1.00, 1.02	0.000 ^g^
		Fiber Residual	−0.03	0.97	0.01	0.95, 0.99	0.007 ^g^
		Sodium Residual	0.11	1.12	0.01	1.01, 1.15	0.000 ^g^
		Calories	0.00	1.00	0.00	1.00, 1.00	0.000 ^g^
**Model 2**	**Carbohydrates**	Fat	−0.00	0.99	0.00	0.99, 1.00	0.546
		Protein	−0.01	0.99	0.00	0.98, 1.00	0.003 ^g^
		Fiber Residual	−0.03	0.97	0.01	0.95, 0.99	0.011 ^g^
		Sodium Residual	0.11	1.11	0.01	1.09, 1.14	0.000 ^g^
		Calories	0.00	1.00	0.00	1.00, 1.00	0.000 ^g^
**Model 3**	**Fat**	Protein	−0.01	0.99	0.00	0.98, 1.00	0.031
		Carbohydrates	0.00	1.00	0.00	0.99, 1.01	0.256
		Fiber Residual	−0.03	0.97	0.01	0.95, 0.99	0.006 ^g^
		Sodium Residual	0.11	1.12	0.01	1.09, 1.14	0.000 ^g^
		Calories	0.00	1.00	0.00	1.00, 1.00	0.000 ^g^
**Across Perceived Healthy Food & Drink Categories**							
	**Excluded**	Fat	−0.00	0.99	0.01	0.99, 1.01	0.833
**Model 1a**	**Protein**	Carbohydrates	−0.00	0.99	0.01	0.99, 1.01	0.799
		Fiber	−0.02	0.98	0.01	0.96, 1.00	0.092
		Sodium	0.08	1.08	0.01	1.06, 1.12	0.000 ^g^
		Healthy Calories	0.00	1.00	0.00	1.00, 1.00	0.184
**Model 2a**	**Carbohydrates**	Total Fat	0.00	1.00	0.00	0.99, 1.01	0.992
		Protein	0.00	1.00	0.01	0.99, 1.01	0.751
		Fiber	−0.02	0.98	0.01	0.96, 1.00	0.067
		Sodium	0.08	1.08	0.01	1.06, 1.11	0.000 ^g^
		Healthy Calories	0.00	1.00	0.00	1.00, 1.00	0.184
**Model 3a**	**Fat**	Protein	0.00	1.00	0.01	0.99, 1.01	0.776
		Carbohydrates	0.00	1.00	0.00	0.99, 1.01	0.986
		Fiber	−0.02	0.99	0.01	0.96, 1.00	0.080
		Sodium	0.08	1.09	0.01	1.06, 1.11	0.000 ^g^
		Healthy Calories	0.00	1.00	0.00	1.00, 1.00	0.183
**Across Perceived Unhealthy Food & Drink Categories**							
	**Excluded**	Fat	0.04	1.04	0.01	1.02, 1.06	0.000 ^g^
**Model 1b**	**Protein**	Carbohydrates	0.04	1.04	0.01	1.02, 1.05	0.000 ^g^
		Fiber	−0.00	0.99	0.01	0.98, 1.02	0.825
		Sodium	0.11	1.13	0.01	1.01, 1.16	0.000 ^g^
		Unhealthy Calories	0.00	1.00	0.00	1.001, 1.002	0.000 ^g^
**Model 2b**	**Carbohydrates**	Fat	−0.00	0.99	0.00	0.99, 1.00	0.469
		Protein	−0.04	0.97	0.01	0.95, 0.99	0.001 ^g^
		Fiber	−0.01	0.99	0.00	0.97, 1.01	0.374
		Sodium	0.12	1.12	0.02	1.09, 1.16	0.000 ^g^
		Unhealthy Calories	0.00	1.00	0.00	1.00, 1.00	0.000 ^g^
**Model 3b**	**Fat**	Protein	−0.03	0.97	0.01	0.95, 0.99	0.004 ^g^
		Carbohydrates	0.00	1.00	0.00	0.99, 1.01	0.214
		Fiber	−0.01	0.99	0.01	0.97, 1.01	0.380
		Sodium	0.12	1.12	0.02	1.09, 1.16	0.000 ^g^
		Unhealthy Calories	0.00	1.00	0.00	1.00, 1.00	0.000 ^g^

^a^Models adjusted for household size, head(s) of household BMI, maximum age for head(s) of household, self-identified race/ethnicity of household, maximum education attainment for head(s) of household, household income, marital status, physical activity, year of data collection, and average total calories per capita available from categories of packaged foods and drinks perceived to be healthy and unhealthy in the home each month

^b^Nutrient residuals for energy bearing nutrients computed using linear regression: kcal of each nutrient over all packaged foods and drinks purchased per month was regressed on the average total calories over all packaged foods and beverages purchased per month. Nutrient residuals are independent of total calories. Point estimates for nutrient residuals reflect a 100-kcal per capita increase. For perceived healthy and unhealthy food and drink categories, the same methods described above were used, separately for healthy and unhealthy categories

^c^Nutrient residuals for sodium and fiber computed using linear regression: grams (or milligrams) of each nutrient over all packaged foods and drinks purchased per month was regressed on the average total calories over all packaged foods and drinks purchased per month. Nutrient residuals are independent of total calories. Point estimates for nutrient residuals reflect an increase per each 0.5 standard deviation per each residual. For sodium and fiber from perceived healthy and unhealthy food and drink categories, the same methods described above were used, separately for healthy and unhealthy categories

^d^13 food categories include: CSDs, cereals, crackers, ice cream, milk, juices, salty snacks, soup, cheese, cookies, frozen dinners, processed meats, yogurt

^e^Perceived Healthy = Cereal, cheese, crackers, juices, milk, soup, and yogurt

^f^Perceived Unhealthy = cookies, CSDs, ice cream, frozen dinners, processed meats, and salty snacks

^g^ Statistically significant using Holm’s step-down approach for multiple comparisons

### Fully adjusted models using quality nutritional metric

Table 4 presents results from the fully adjusted logistic regression model evaluating the associations between average per capita grams of each nutrient per serving per month across the packaged food and drinks and DRCMM household status. This model evaluates the energy-density of packaged food and drink purchases. On average, purchases across all 13 packaged food and drinks were less energy-dense among DRCMM households than non-DRCMM household when considering each energy-bearing nutrient (Table 4, model 1, *p* < 0.001, for all). For example, the odds of being a DRCMM household decreased by 17% (OR: 0.83; 95% CI: 0.79, 0.86; *p* = 0.000) for each additional per capita gram of protein per serving per month among the packaged foods and drinks in the home; a finding consistent with the previous model demonstrating that non-DRCMM households averaged more per capita grams of protein from the food and drinks purchases. The associations between per capita grams of carbohydrate and fat per serving per month were less pronounced than for protein, where the odds of being a DRCMM household decreased by 6% (OR: 0.94; 95% CI: 0.93, 0.95; *p* = 0.000) and 9.0% (OR: 0.91; 95% CI: 0.88, 0.95; p = 0.000) for each additional per capita gram of carbohydrates and fat, respectively, per serving per month among the packaged foods and drinks in the home.

**Table 4:** Adjusted^a^ odds ratios of diet-related cardiometabolic multi-morbid households relative to monthly weighed per capita nutrients per serving, controlling for calories per capita from food & drinks^b^ and when grouped into perceived healthy^c^ and unhealthy^d^

	Coefficient	Odds Ratio	Standard Error	95% Confidence Interval	*P* Value^**e**^
**Model 1: Across all 13 Food & Drink Categories**					
**Protein (g/serving)**	− 0.19	0.83	0.02	0.79, 0.87	0.000^**e**^
**Carbohydrates (g/serving)**	−0.07	0.94	0.01	0.93, 0.95	0.000^**e**^
**Fat (g/serving)**	−0.09	0.91	0.02	0.88, 0.95	0.000^**e**^
**Fiber (g/serving)**	0.05	1.05	0.07	0.92, 1.21	0.450
**Sodium (mg/serving)**	0.00	1.00	0.00	1.00, 1.00	0.000^**e**^
**Calories (kcal/capita- 0.5 SD Kcal)**	0.06	1.06	0.01	1.04, 1.08	0.000^**e**^
**Model 2: Perceived Healthy Food & Drinks**					
**Protein (g/serving)**	−0.07	0.93	0.03	0.88, 0.99	0.023^**e**^
**Carbohydrates (g/serving)**	−0.07	0.93	0.01	0.92, 0.95	0.000^**e**^
**Fat (g/serving)**	−0.16	0.85	0.03	0.80, 0.91	0.000^**e**^
**Fiber (g/serving)**	0.08	1.08	0.09	0.91, 1.29	0.373
**Sodium (mg/serving)**	0.00	1.00	0.00	1.00, 1.00	0.000^**e**^
**Calories Healthy (kcal/capita- 0.5 SD Kcal)**	0.00	1.00	0.00	1.00, 1.00	0.000^**e**^
**Model 3: Perceived Unhealthy Food & Drinks**					
**Protein (g/serving)**	−0.32	0.73	0.07	0.64, 0.83	0.000^**e**^
**Carbohydrates (g/serving)**	−0.04	0.96	0.01	0.95, 0.98	0.000^**e**^
**Fat (g/serving)**	−0.08	0.92	0.03	0.87, 0.97	0.004^**e**^
**Fiber (g/serving)**	0.00	1.00	0.18	0.70, 1.43	0.991
**Sodium (mg/serving)**	0.01	1.01	0.00	1.01, 1.01	0.000^**e**^
**Calories Unhealthy (kcal/capita- 0.5 SD kcal)**	0.00	1.00	0.00	1.02, 1.00	0.000^**e**^

*kcal* Kilocalorie, *SD* Standard deviation

^a^ Models adjusted for household size, head(s) of household BMI, maximum age for head(s) of household, self-identified race/ethnicity of household, maximum education attainment for head(s) of household, household income, marital status, physical activity, year of data collection, and average total calories per capita available from packaged food and drinks in the home each month

^b^Food and drink categories include 13 categories related to diet: CSDs, cereals, crackers, ice cream, milk, juices, salty snacks, soup, cheese, cookies, frozen dinners, processed meats, yogurt

^c^ Perceived Healthy = Cereal, cheese, crackers, milk, soup, and yogurt

^d^ Perceived Unhealthy = cookies, ice cream, frozen dinners, processed meats, and salty snacks

^e^ Statistically significant using Holms step-down correction applied for multiple comparison across 13 food and drink categories

When considering perceived healthy and perceived unhealthy categories separately, results differed for the various nutrients per serving per month. The lower per capita protein per serving per month for DRCMM households compared with non-DRCMM households was more pronounced for perceived unhealthy categories (OR: 0.73; 95% CI: 0.64, 0.83; *p* = 0.000) than perceived healthy categories (OR: 0.93; 95% CI: 0.88, 0.99; *p* = 0.023). However, the lower per capita fat per serving per month for DRCMM households was more pronounced for perceived healthy categories (OR: 0.85; 95% CI: 0.80, 0.91; p = 0.000) than perceived unhealthy categories (OR: 0.92; 95% CI: 0.87, 0.97; *p* = 0.004).

### Sensitivity analysis of fully adjusted models using quality nutritional metric

Table 5 includes results from the sensitivity analysis of the fully adjusted logistic regression model evaluating the associations between average per capita grams of each nutrient per serving per month across the packaged food and drinks, excluding CDSs and juice, and DRCMM household status. This model evaluates energy-density of packaged food and drink, excluding CSDs and juices. When CSDs and juices were removed, packaged food and drink purchases from DRCMM households contained fewer per capita grams of protein and carbohydrates per serving per month when compared to their counterparts (*p* < 0.001 for both). These findings partially confirm results from the analysis including CSDs and juices, and support the conclusion that on average, purchases from DRCMM households contained fewer per capita grams of protein and carbohydrates per serving per month. When CSDs and juices were removed, per capita grams of fat per serving per month from all packaged food and drink purchases were not different across households with and without DRCMM (*p* = 0.091). Moreover, DRCMM households purchased more per capita fat per serving per month from perceived unhealthy categories when CSDs and juices were removed, however, the association was not statistically significant (*p* = 0.179). While statistically insignificant, these findings are consistent with results from the first fully adjusted model where DRCMM households purchased more fat in lieu of protein.

**Table 5:** Adjusted^a^ odds ratios of diet-related cardiometabolic multimorbid households relative to monthly weighed per capita nutrients per serving, controlling for calories per capita from all food & drinks^b^ and when grouped into perceived healthy^c^ and unhealthy^d^, excluding carbonated soft drinks and juices

	Coefficient	Odds Ratio	Standard Error	95% Confidence Interval	***P*** Value^**e**^
**Model 1: Across all 11 Food & Drink Categories**					
**Protein (g/serving)**	− 0.11	0.90	0.02	0.85, 0.95	0.000 ^e^
**Carbohydrates (g/serving)**	− 0.04	0.96	0.01	0.95, 0.98	0.000 ^e^
**Fat (g/serving)**	−0.03	0.97	0.02	0.94, 1.00	0.091
**Fiber (g/serving)**	−0.02	0.98	0.07	0.85, 1.11	0.794
**Sodium (mg/serving)**	0.00	1.00	0.00	1.00, 1.00	0.000 ^e^
**Calories (kcal/capita- 0.5 SD Kcal)**	0.05	1.05	0.01	1.03, 1.07	0.000 ^e^
**Model 2: Perceived Healthy Food & Drinks**					
**Protein (g/serving)**	−0.08	0.92	0.03	0.87, 0.97	0.001 ^e^
**Carbohydrates (g/serving)**	−0.01	0.99	0.01	0.97, 1.01	0.150
**Fat (g/serving)**	−0.06	0.95	0.03	0.89, 0.99	0.036
**Fiber (g/serving)**	−0.20	0.82	0.08	0.66, 0.98	0.015 ^e^
**Sodium (mg/serving)**	0.00	1.00	0.00	1.00, 1.00	0.000 ^e^
**Calories Healthy (kcal/capita- 0.5 SD Kcal)**	0.00	1.00	0.00	1.00, 1.00	0.000 ^e^
**Model 3: Perceived Unhealthy Food & Drinks**					
**Protein (g/serving)**	−0.11	0.89	0.05	0.80, 0.99	0.021
**Carbohydrates (g/serving)**	−0.04	0.96	0.01	0.94, 0.98	0.000 ^e^
**Fat (g/serving)**	0.03	1.03	0.02	0.98, 1.08	0.197
**Fiber (g/serving)**	0.26	1.30	0.16	0.98, 1.62	0.105
**Sodium (mg/serving)**	0.00	1.00	0.00	1.00, 1.00	0.000 ^e^
**Calories Unhealthy (kcal/capita- 0.5 SD Kcal)**	0.00	1.00	0.00	1.00, 1.00	0.000 ^e^

*kcal* Kilocalorie, *SD* Standard deviation

^a^ Models adjusted for household size, head(s) of household BMI, maximum age for head(s) of household, self-identified race/ethnicity of household, maximum education attainment for head(s) of household, household income, marital status, physical activity, year of data collection, and average total calories per capita available from packaged food and drinks in the home each month

^b^13 food categories include: Cereals, crackers, ice cream, milk, salty snacks, soup, cheese, cookies, frozen dinners, processed meats, yogurt, baked goods/desserts, candy/confectionary

^c^ Perceived Healthy = Cereal, cheese, crackers, milk, soup, and yogurt

^d^ Perceived Unhealthy = cookies, ice cream, frozen dinners, processed meats, salty snacks, baked goods/desserts, candy/confectionary

^e^ Statistically significant using Holms step-down correction applied for multiple comparison across 11 food and drink categories

### Differences across food and drink categories

To better understand differences in purchases of individual packaged food and drink categories among DRCMM households, exploratory analysis was performed individually for each of the 13 food and drink categories. Results from this analysis are available in the online supplemental. Additional file 3 provides the average nutrient and calorie content per serving per month for each food and drink category. Food and drink categories with the highest fat content per serving per month were frozen dinners and processed meats, while those with the highest carbohydrate content per serving per month were cereals and yogurt. Across food and drink categories, DRCMM households purchased more total calories per capita per month from 6 food and drink categories: CSDs, cookies, crackers, ice cream, processed meats, and soup, (Additional file 4). Findings for total servings per capita per month were similar (Additional file 5).

### Differences within food and drink categories

When considering energy-density, on average, DRCMM households purchased less energy-dense (expressed as total per capita calories per serving per month) CSDs, frozen dinners, ice cream, juices, and yogurt than non-DRCMM households (Additional file 6). Among these less-energy dense items, DRCMM households also purchased more servings and calories from CSDs, ice cream, and juices (Additional file 6). The low energy-dense versions of CSDs and juices likely represents diet drinks. Results from sensitivity analysis show that purchases from DRCMM households contained fewer grams of protein and carbohydrates per serving per month, even when removing CSDs and juices. This suggests the low nutrient-dense purchases seen among DRCMM households were not limited to low nutrient-dense versions of CSDs and juices. Instead, DRCMM households on average purchased food and drinks with fewer grams of protein and carbohydrates per serving per month, likely driven by low nutrient-dense versions of frozen dinners, ice cream, and yogurt. In contrast, DRCMM households purchased more energy-dense (expressed as calories per serving) processed meats and salty snacks than non-DRCMM households (Additional file 6).

## Discussion

In this cross-sectional study, data from 22,750 households were used to investigate the associations between packaged food and drink purchases and DRCMM. First, DRCMM households purchased greater *quantities* of all food and drinks, especially perceived unhealthy items. Second, when evaluating total energy from packaged food and drinks in the home, DRCMM households purchased fat and carbohydrates in lieu of protein and this trend was driven by perceived unhealthy categories. Third, when considering the nutrient-density of all food and drinks purchased for the home, DRCMM households contained less nutrient-dense food and drinks. When removing CSDs and juices, a customary method when evaluating energy or nutrient-density, purchases from DRCMM households contained fewer per capita grams of protein and carbohydrates per serving [26]. Differences in total energy and nutrient-density of food and drinks purchased for the home reflect differences in the modelling techniques employed in this study. Models evaluating energy or nutrient-density capture servings and models evaluating total energy do not. While differences in the total energy and nutrient-density of packaged food and drinks were observed, the greater availability of calories and nutrients per capita in DRCMM homes was largely driven by increased *quantities* of food and drinks. Results were independent of household and household head(s) characteristics, including BMI, age, race, income, education, marital status, and physical activity. Importantly, these results also controlled for the expected caloric intake of the household based on family size and the age of each family member. While the design of the study limits conclusions on directionality, the discussion identifies areas in need of intervention for DRCMM households.

Increased *quantities* of purchases provided DRCMM households with more total nutrients and calories. Prior research suggests the greater availability of food leads to increased consumption [27]. Moreover, DRCMM households purchased more fat, which provides more energy-density when compared to protein and carbohydrates [24]. The additional fat purchased by DRCMM households was driven by purchases from perceived unhealthy categories. It is generally well-accepted that increased consumption of unhealthy processed foods is a risk factor for obesity and diet-related non-communicable diseases [13, 14, 28–30]. The added sugar, fat, and sodium along with additional processing techniques create food and drink products with poor nutritional quality and these items are hypothesized to convey adverse health effects including cardiovascular disease, obesity, and type 2 diabetes [16, 31, 32]. Broadly speaking, dietary guidelines typically recommend reducing consumption of processed food and drinks [12, 13]. Despite these recommendations, increased *quantities* of packaged food and drinks, especially among perceived unhealthy items, provided DRCMM households with greater availability of nutrients and calories per capita. To that end, findings from this study underscore the importance of reducing the availability of unhealthy packaged food and drinks within the home to promote a healthy lifestyle for those with DRCMM.

While DRCMM households had more fat and total calories in their homes, on average the nutrient-density of their purchases was lower than households without DRCMM. In other words, purchases from DRCMM households contained fewer per capita grams of protein, carbohydrates, and fat per serving when considering all food and drink categories. When CSDs and juices were removed, these results remained consistent for protein and carbohydrates. Consistent with existing literature, excluding CSDs and juices was important to evaluating energy-density, as the contribution of many servings, relative to nutrients and calories from CSDs and juices, skewed the energy-density when averaged across all 13 packaged food and drinks [26]. Results from the sensitivity analysis suggests DRCMM households may isolate their low energy-dense purchases to specific food and drink items. For example, DRCMM households purchased CSDs, juices, frozen dinners, ice cream, and yogurt with lower energy-density when compared to their counterparts. However, no differences in nutrient-density were observed for cheese, cookies, and crackers. While DRCMM households purchased some food and drink with lower total energy-density, they also purchased greater quantities, such that benefits from purchasing less energy-dense items may be offset by purchasing more of them. Findings from this study suggest portion size among packaged food and drinks, may be another point of intervention to encourage healthy lifestyle practices among those with DRCMM.

Additional patterns in food and drink purchases were observed among DRCMM households. For example, DRCMM households purchased greater *quantities* of cookies, and processed meats, which generated more total calories from both items. Cookies and processed meats are typically considered ultra-processed foods with added fat, sugar, and sodium [12, 29, 30, 33] and other additives to enhance taste, appearance and texture. The consumption of processed, especially ultra-processed food and drinks, is an emerging topic in nutrition science and has recently been associated with numerous adverse health outcomes, including cardiovascular disease [32, 34], cancer [16], and metabolic disorders [31, 33]. Moreover, a greater intake of processed meat alone is well-documented risk factor for obesity, coronary heart disease, and diabetes [35, 36]. In a prior study using a comparative risk assessment model, researchers determined the largest number of estimated diet-related cardiometabolic deaths were related to processed meat intake [35]. As it relates to multi-morbidity, a cross-sectional study conducted in the Netherlands determined adults with multi-morbidity consumed more meat and snacks, consistent with results from our study [1]. In addition, prior research found that diets low in processed food and drinks protect against coronary heart disease, stroke, and diabetes [37, 38]. Moreover, dietary recommendations to prevent chronic disease support diets low in saturated fat, trans fats, sodium, and added sugar [39, 40]. Given prior research and recommendations to limit processed and ultra-processed items, interventions aimed at reducing the quantity of processed meats and cookies among DRCMM populations may be an appropriate step toward promoting healthy dietary practices.

## Limitations

This study is not without limitations. First and foremost, it is a cross-sectional study and cannot speak to a causal effect of packaged food and drinks on the development of multi-morbidity. While a major limitation, the nascency of this research topic warrants an exploration study. Second, the data track food and drink purchases of the household and cannot measure consumption of the household or of individual household members. However, food and drink purchases are frequently used to measure the food environment of households and are generally considered reasonable estimates of food and diet [41–44]. Third, since this study did not include fresh foods, the findings are limited to packaged food and drinks. However, packaged food and drinks reasonably reflect most diet choices, as the majority of calories consumed in the U.S. are derived from moderate or highly processed packaged items [12, 13, 15]. Fourth, another limitation relates to the specific food and drink categories used in this study. We included the 13 largest categories but there are other energy-dense packaged foods, e.g., baked goods, desserts, and candy/confectionary. These categories were not included in the main analysis because nutrient data were available for a substantially smaller percentage of the SKUs in these categories. Still, all the analysis was repeated with the inclusion of baked goods/desserts and candy/confectionary categories and results are available in Additional files 7–10. Results were largely consistent with those presented in the main manuscript, confirming their robustness. However, the association of per capita fiber and fat per serving from perceived unhealthy became non-significant with the inclusion of baked goods/dessert and candy/confectionary categories. As such, the 2 added categories may alter measurements for these 2 macronutrients and caution is warranted when interpreting their associations. Finally, the data used in this study were collected over 10 years ago. Although the data are older, the 13 food and drink categories used in this analysis are still prominent today [45]. While the specific food and drink items comprising these 13 categories may experience some fluctuation over time, as a group, packaged food and drinks have remained a consistent target for nutritional and policy intervention [12, 13, 46, 47]. As such, findings from this study, can still offer worthwhile guidance.

## Conclusion

This study identified differences in packaged food and drink purchases among households with DRCMM. Overall, DRCMM households purchased more packaged food and drinks per capita, especially unhealthy items such as processed meats and cookies. While DRCMM households purchased some food and drinks with lower nutrient-density per serving, they purchased more calories from fat and carbohydrates, and greater amounts of sodium from their packaged food and drink purchases, supporting that overall, the home food environment may not best support a healthy dietary pattern recommended to manage DRCMM. Findings support that interventions to help households with DRCMM improve the nutritional quality of the food home environment are warranted.

## Supplementary Information

**Additional file 1.** Covariate Categories. Details on the operationalization of covariate variables.

**Additional file 2.** Outcome, Main Exposures, and Covariate Variable Operationalization. Details on the operationalization of variables used in study analysis.

**Additional file 3.** Average Nutrient Content Per Serving* with Standard Deviations from 13 Food and Drink Categories. Supplemental analysis on average nutrient content per serving from 13 food and drink categories used in this study analysis.

**Additional file 4.** One-Year Monthly Average Weighed Per Capita Calories (kcal) Per Food Group, Stratified by Diet-Related Cardiometabolic Multi-Morbid Households. Supplemental analysis the on the average calories per capita for each food and drink category grouping.

**Additional file 5.** One-Year Monthly Average Weighed Per Capita Serving (#) Per Food Group, Stratified by Diet-Related Cardiometabolic Multi-Morbid Households. Supplemental analysis the on average calories per serving for each food and drink category grouping.

**Additional file 6.** One-Year Monthly Average Weighed Per Capita Calories (kcal) Per Serving Per Food Group, Stratified by Diet-Related Cardiometabolic Multi-Morbid Households. Supplemental analysis the on the average per capita calories per serving from each food and drink category grouping.

**Additional file 7.** Unadjusted associations for 3 nutritional profile metrics across all food and drink categories^a^ and grouped into those perceived as healthy^b^ and unhealthy^c^, stratified by diet-related cardiometabolic multi-morbid household status, when including baked goods/desserts and candy/confectionary. Supplemental analysis re-running unadjusted associations with the inclusion of baked goods/desserts and candy/confectionary foods.

**Additional file 8.** Regression coefficients and adjusted^a^ odds ratios of diet-related cardiometabolic multi-morbid households relative to 100 kcal per capita, per month, estimated by various residual models^b^, for purchases from 3 calorie sources, fiber, sodium, and calories from all food & drinks^c^ and when grouped into perceived healthy^d^ & unhealthy^e^ when including baked goods/desserts and candy/confectionary items. Supplemental analysis re-running multiple variable logistic regression substitution models using residuals with the inclusion of baked goods/desserts and candy/confectionary food items.

**Additional file 9.** Adjusted^a^ odds ratios of diet-related cardiometabolic multi-morbid households relative to monthly weighed per capita nutrients per serving, controlling for calories per capita from food & drinks^b^ and when grouped into perceived healthy^c^ and unhealthy^d^ with inclusion of baked goods/desserts and candy/confectionary. Supplemental analysis re-running multiple variable logistic regression model using per serving (quality) variables with the inclusion of baked goods/desserts and candy/confectionary food items.

**Additional file 10.** Adjusted^a^ odds ratios of diet-related cardiometabolic multimorbid households relative to monthly weighed per capita nutrients per serving, controlling for calories per capita from all food & drinks^b^ and when grouped into perceived healthy^c^ and unhealthy^d^, excluding carbonated soft drinks and juices and including baked goods/desserts and candy/confectionary. Supplemental analysis re-running multiple variable logistic regression model using per serving (quality) variables without carbonated soft drink and juices but with the inclusion of baked goods/desserts and candy/confectionary food items.

## Data Availability

The data that support the findings of this study were made available from the market research firm Information Resources Inc. (formally Symphony IRI). Data were made available for research purposes pursuant to a non-disclosure agreement. Code and protocol materials are available upon request to the corresponding author, Iben M. Ricket.
