# Supplementary material for: Quantifying differences in packaged food and drink purchases among households with diet-related cardiometabolic multi-morbidity: a cross-sectional analysis
Source: BMC Public Health. 2022 Nov 17;22:2101. doi: 10.1186/s12889-022-14626-3 (PMC9670385; doi:10.1186/s12889-022-14626-3)
Supplement: Supplementary file 1 — Additional file 1. Covariate Categories. Details on the operationalization of covariate variables. [file 12889_2022_14626_MOESM1_ESM.pdf]

Additional File 1. Covariate Categories

Covariate	Covariate Categorization
BMI	Kg/m2 (self-reported from height & weight)
Physical Activity	Most days<br>Somedays<br>Rarely/never
Family Size	One Person<br>Two People<br>Three People<br>Four People<br>Five People<br>Six People<br>Seven People<br>Eight or more People
Income	\$00,000 TO \$ 9,999 PER YR<br>\$10,000 TO \$11,999 PER YR<br>\$12,000 TO \$14,999 PER YR<br>\$15,000 TO \$19,999 PER YR<br>\$20,000 TO \$24,999 PER YR<br>\$25,000 TO \$34,999 PER YR<br>\$35,000 TO \$44,999 PER YR<br>\$45,000 TO \$54,999 PER YR<br>\$55,000 TO \$64,999 PER YR<br>\$65,000 TO \$74,999 PER YR<br>\$75,000 TO \$99,999 PER YR<br>\$100,000 AND GREATER PER YR
Race	WHITE<br>BLACK<br>HISPANIC<br>ASIAN<br>OTHER
Head of Household Age	18 - 29<br>30 - 34<br>35 - 44<br>45 - 54<br>55 - 64<br>65 & OVER
Head of Household Education	SOME GRADE SCHOOL OR LESS<br>COMPLETED GRADE SCHOOL<br>SOME HIGH SCHOOL<br>GRADUATED HIGH SCHOOL<br>TECHNICAL SCHOOL<br>SOME COLLEGE<br>GRADUATED FROM COLLEGE<br>POST GRADUATE WORK
Head of Household Martial Status	SINGLE<br>MARRIED<br>DIVORCED<br>WIDOWED<br>SEPARATED
