# Supplementary material for: Quantifying differences in packaged food and drink purchases among households with diet-related cardiometabolic multi-morbidity: a cross-sectional analysis
Source: BMC Public Health. 2022 Nov 17;22:2101. doi: 10.1186/s12889-022-14626-3 (PMC9670385; doi:10.1186/s12889-022-14626-3)
Supplement: Supplementary file 2 — Additional file 2. Outcome, Main Exposures, and Covariate Variable Operationalization. Details on the operationalization of variables used in study analysis. [file 12889_2022_14626_MOESM2_ESM.pdf]

Additional File 2. Outcome, Main Exposures, and Covariate Variable Operationalization

	Variable	Definition
**Outcome**	Diet-related cardiometabolic multi-morbidity	Household head(s) self reporting 2 or more diet-related cardiometabolic chronic conditions
		Diet-related cardiometabolic chronic conditions include: angina, atherosclerosis, hyperlipidemia, congestive heart failure, diabetes, myocardial infarction, hypertension, or stroke
**Main Exposures**	Weighed per capita	Main exposure variables controlled for household size and age composition of household members
		Household members weighted by age to account for the expected daily caloric requirements relative to the recommended 2,000 calorie/day for adults. Age 13 years + were given a weight of 1, age 6-12 years were given a weight of 0.75, and age 2-5 years were given a weight of 0.575.
	Packaged Food & Drink *Quantity*	Servings per capita
		Servings defined as total volume of item/serving size of item, with serving size as content consmed typically per eating occasion
	Packaged Food & Drink *Quality*	Calories, protein, carbohydrates, fat, fiber, and sodium, expressed as weighed per capita nutrients per serving
		Servings defined as total volume of item/serving size of item, with serving size as content consmed typically per eating occasion
		Calories, protein, carbohydrates, fat, fiber, and sodium calculated from all purchases across all 13 food and drink cateogries
	Packaged Food & Drink *Quantity*Quality*	Calories, protein, carbohydrates, fat, fiber, and sodium weighed per capita
		Calories, protein, carbohydrates, fat, fiber, and sodium calculated from total monthly purchases across all 13 food and drink cateogries
**Covariates**	BMI	BMI of household head
	Age	Age of household head
	Education	Education of household head, categories include: some high school, high school diploma, some college, college degree, post graduate work or technical school
	Physical Activity	Weekly physical activity of household head, categories include: rarley/never, somedays, most days (reference is most days)
	Race	White, Black, Hispanic, Asian, Other
	Income	Household income in dollars, categories in 10K increments from $0-9,999 (reference) to $100,000+
	Family Size	Number people living in household, categories from 1 (reference) to 8+ people
	Marital Status	Household head marital status, categories include married, divorced, separated, widowed
	Survey Year	Year of household data collection, categories include 2006 (reference), 2007, and 2008
