# Supplementary material for: Quantifying differences in packaged food and drink purchases among households with diet-related cardiometabolic multi-morbidity: a cross-sectional analysis
Source: BMC Public Health. 2022 Nov 17;22:2101. doi: 10.1186/s12889-022-14626-3 (PMC9670385; doi:10.1186/s12889-022-14626-3)
Supplement: Supplementary file 3 — Additional file 3. Average Nutrient Content Per Serving* with Standard Deviations from 13 Food and Drink Categories. Supplemental analysis on average nutrient content per serving from 13 food and drink categories used in this study analysis. [file 12889_2022_14626_MOESM3_ESM.pdf]

Additional File 3. Average Nutrient Content Per Serving* with Standard Deviations from 13 Food and Drink Categories

**Food Category**	**Calories (kcal)**	**Protein (g)**	**Carbohydrates (g)**	**Fat (g)**	**Fiber (g)**	**Sodium (mg)**
Perceived Healthy						
Cereals	202.6 (15.3)	4.3 (1.6)	43.9 (3.5)	2.1 (1.0)	3.8 (1.6)	270.7 (73.2)
Cheese	98.2 (10.3)	6.6 (0.8)	1.1 (0.6)	7.4 (1.1)	0.00 (0.0)	262.4 (62.7)
Crackers	135.4 (14.3)	2.4 (0.6)	20.0 (2.3)	5.1 (1.3)	0.6 (0.7)	260.5 (50.3)
Juices	100.2 (27.1)	0.70 (0.6)	24.3 (6.7)	0.1 (0.7)	0.2 (0.4)	71.5 (100.3)
Milk	122.9 (24.8)	8.1 (0.6)	13.4 (2.7)	3.9 (2.5)	0.0 (0.2)	132.1 (16.1)
Soup	216.8 (196.9)	6.8 (4.8)	30.4 (31.0)	7.0 (7.0)	2.6 (2.5)	2090.2 (3291.0)
Yogurt	172.5 (41.1)	7.7 (1.5)	31.1 (8.0)	1.7 (1.5)	0.4 (0.8)	121.2 (21.7)
Perceived Unhealthy						
CSDs	60.5 (45.1)	0.00 (0.0)	16.3 (12.1)	0.00 (0.0)	0.0 (0.0)	29.2 (10.5)
Cookies	138.1 (12.2)	1.3 (0.4)	20.4 (1.7)	5.9 (1.2)	0.4 (0.4)	108.0 (67.9)
Frozen Dinners	262.0 (71.4)	11.3 (2.3)	29.7 (7.8)	10.6 (3.9)	2.2 (0.6)	603.1 (138.6)
Ice Cream	149.4 (30.0)	2.6 (0.6)	19.5 (3.2)	7.0 (2.5)	0.4 (0.5)	56.9 (13.6)
Processed Meats	129.6 (30.6)	7.8 (2.1)	1.6 (1.1)	10.1 (3.2)	0.0 (0.2)	532.4 (154.7)
Salty Snacks	148.0 (20.0)	2.2 (0.7)	17.9 (2.3)	7.7 (2.4)	1.0 (0.3)	221.1 (82.4)
*Expressed as one-year monthly average weighed per capita nutrient per serving, average (standard deviation)						
