# Supplementary material for: Quantifying differences in packaged food and drink purchases among households with diet-related cardiometabolic multi-morbidity: a cross-sectional analysis
Source: BMC Public Health. 2022 Nov 17;22:2101. doi: 10.1186/s12889-022-14626-3 (PMC9670385; doi:10.1186/s12889-022-14626-3)
Supplement: Supplementary file 4 — Additional file 4. One-Year Monthly Average Weighed Per Capita Calories (kcal) Per Food Group, Stratified by Diet-Related Cardiometabolic Multi-Morbid Households. Supplemental analysis the on the average calories per capita for each food and drink category grouping. [file 12889_2022_14626_MOESM4_ESM.pdf]

Additional File 4. One-Year Monthly Average Weighed Per Capita Calories (kcal) Per Food Group, Stratified by Diet-Related Cardiometabolic Multi-Morbid Households

	Non-Diet Related Multi-Morbid Household mean (SD)	*Diet Related Multi-Morbid Household mean (SD)*	*P-value**
**N Households**	18955	3795	
**CSDs (kcal/capita)**	1394.6 (2339.0)	1531.7 (2887.8)	0.002**
**Cereals (kcal/capita)**	1810.8 (1770.8)	1756.0 (1909.8)	0.093
**Cheese (kcal/capita)**	1377.0 (1111.5)	1375.01 (1479.5)	0.927
**Cookies (kcal/capita)**	1260.2 (1573.4)	1524.1 (1787.5)	<0.001**
**Crackers (kcal/capita)**	1170.0 (1170.7)	1327.3 (1783.1)	<0.001*
**Frozen Dinners (kcal/capita)**	1387.5 (1516.7)	1348.6 (1683.4)	0.168
**Ice Cream (kcal/capita)**	1150.1 (1456.0)	1276.9 (1551.8)	<0.001**
**Juices (kcal/capita)**	1003.2 (1161.0)	1019.4 (1314.5)	0.449
**Milk (kcal/capita)**	2235.4 (2076.6)	2331.6 (2380.8)	0.012
**Processed Meats (kcal/capita)**	1325.7 (1212.8)	1693.5 (1798.2)	<0.001**
**Salty Snacks (kcal/capita)**	2206.0 (2000.7)	2218.0 (2138.2)	0.741
**Soup (kcal/capita)**	443.8 (536.4)	519.0 (816.6)	<0.001**
**Yogurt (kcal/capita)**	414.9 (576.7)	370.3 (548.9)	<0.001**
*P-value calculated using Chi square and 2-sample T-test for categorical and continuous variables, respectively			
**Statistically significant using Holms step-down correction applied for multiple comparison across 13 food and drink categories			
