# Supplementary material for: Quantifying differences in packaged food and drink purchases among households with diet-related cardiometabolic multi-morbidity: a cross-sectional analysis
Source: BMC Public Health. 2022 Nov 17;22:2101. doi: 10.1186/s12889-022-14626-3 (PMC9670385; doi:10.1186/s12889-022-14626-3)
Supplement: Supplementary file 5 — Additional file 5. One-Year Monthly Average Weighed Per Capita Serving (#) Per Food Group, Stratified by Diet-Related Cardiometabolic Multi-Morbid Households. Supplemental analysis the on average calories per serving for each food and drink category grouping. [file 12889_2022_14626_MOESM5_ESM.pdf]

Additional File 5. One-Year Monthly Average Weighed Per Capita Serving (#) Per Food Group, Stratified by Diet-Related Cardiometabolic Multi-Morbid Households

	Non-Diet Related Multi-Morbid Household mean (SD)	***Diet Related Multi-Morbid Household mean (SD)***	***P-value****
**N Households**	18955	3795	
**CSDs (servings/capita)**	26.0 (32.9)	33.5 (43.9)	<0.001**
**Cereals (servings/capita)**	8.9 (8.7)	8.7 (9.4)	0.164
**Cheese (servings/capita)**	14.0 (11.2)	14.0 (14.5)	0.989
**Cookies (servings/capita)**	9.1 (11.2)	11.0 (12.7)	<0.001**
**Crackers (servings/capita)**	8.6 (8.4)	9.8 (12.1)	<0.001**
**Frozen Dinners (servings/capita)**	5.5 (6.3)	5.6 (7.4)	0.387
**Ice Cream (servings/capita)**	7.8 (10.0)	8.9 (10.9)	<0.001**
**Juices (servings/capita)**	9.7 (10.8)	10.3 (12.5)	0.004**
**Milk (servings/capita)**	18.7 (17.2)	19.4 (19.9)	0.038
**Processed Meats (servings/capita)**	10.2 (8.9)	12.9 (13.0)	<0.001**
**Salty Snacks (servings/capita)**	14.9 (13.1)	14.9 (14.4)	0.97
**Soup (servings/capita)**	2.4 (2.5)	2.9 (3.1)	<0.001**
**Yogurt (servings/capita)**	2.5 (3.4)	2.3 (3.4)	0.013
*P-value calculated using Chi square and 2-sample T-test for categorical and continuous variables, respectively			
**Statistically significant using Holms step-down correction applied for multiple comparison across 13 food and drink categories			
