# Supplementary material for: Quantifying differences in packaged food and drink purchases among households with diet-related cardiometabolic multi-morbidity: a cross-sectional analysis
Source: BMC Public Health. 2022 Nov 17;22:2101. doi: 10.1186/s12889-022-14626-3 (PMC9670385; doi:10.1186/s12889-022-14626-3)
Supplement: Supplementary file 6 — Additional file 6. One-Year Monthly Average Weighed Per Capita Calories (kcal) Per Serving Per Food Group, Stratified by Diet-Related Cardiometabolic Multi-Morbid Households. Supplemental analysis the on the average per capita calories per serving from each food and drink category grouping. [file 12889_2022_14626_MOESM6_ESM.pdf]

Additional File 6. One-Year Monthly Average Weighed Per Capita Calories (kcal) Per Serving Per Food Group, Stratified by Diet-Related Cardiometabolic Multi-Morbid Households

	Non-Diet Related Multi-Morbid Household mean (SD)	*Diet Related Multi-Morbid Household mean (SD)*	*P-value**
**N Households**	18955	3795	
**CSDs (kcal/serving)**	62.3 (45.2)	51.8 (43.7)	<0.001**
**Cereals (kcal/serving)**	202.7 (13.8)	202.2 (21.5)	0.046
**Cheese (kcal/serving)**	98.2 (10.2)	98.2 (10.7)	0.878
**Cookies (kcal/serving)**	138.0 (12.4)	138.3 (11.5)	0.282
**Crackers (kcal/serving)**	135.4 (14.6)	135.1 (13.1)	0.296
**Frozen Dinners (kcal/serving)**	264.0 (71.3)	251.8 (70.9)	<0.001**
**Ice Cream (kcal/serving)**	149.9 (30.4)	147.3 (27.5)	<0.001**
**Juices (kcal/serving)**	100.8 (26.0)	97.1 (31.7)	<0.001**
**Milk (kcal/serving)**	122.7 (24.9)	123.6 (24.2)	0.045
**Processed Meats (kcal/serving)**	129.3 (31.0)	131.0 (28.5)	0.002**
**Salty Snacks (kcal/serving)**	147.7 (20.0)	149.3 (20.2)	<0.001**
**Soup (kcal/serving)**	218.3 (203.5)	209.4 (159.5)	0.011
**Yogurt (kcal/serving)**	173.3 (40.7)	167.9 (42.8)	<0.001**
*P-value calculated using Chi square and 2-sample T-test for categorical and continuous variables, respectively			
*Statistically significant using Holms step-down correction applied for multiple comparison across 13 food and drink categories			
