# Supplementary material for: Quantifying differences in packaged food and drink purchases among households with diet-related cardiometabolic multi-morbidity: a cross-sectional analysis
Source: BMC Public Health. 2022 Nov 17;22:2101. doi: 10.1186/s12889-022-14626-3 (PMC9670385; doi:10.1186/s12889-022-14626-3)
Supplement: Supplementary file 7 — Additional file 7. Unadjusted associations for 3 nutritional profile metrics across all food and drink categoriesa and grouped into those perceived as healthyb and unhealthyc, stratified by diet-related cardiometabolic multi-morbid household status, when including baked goods/desserts and candy/confectionary. Supplemental analysis re-running unadjusted associations with the inclusion of baked goods/desserts and candy/confectionary foods. [file 12889_2022_14626_MOESM7_ESM.pdf]

Additional File 7. Unadjusted associations for 3 nutritional profile metrics across all food and drink categories[a] and grouped into those perceived as healthy[b] and unhealthy[c], stratified by diet-related cardiometabolic multi-morbid household status, when including baked goods/desserts and candy/confectionary

| **Outcome Status** | *Non-Diet Related Multi-Morbid Household mean (SD)* | *Diet Related Multi-Morbid Household mean (SD)* | *P-value*[d] |
|---|---|---|---|
| **N households** | 18955 | 3795 | |
| **Quantity Nutritional Profile**[e] | | | |
| **Servings (#/capita)** | 182.9 (95.8) | 205.2 (126.9) | 0.00 [h] |
| **Servings Healthy (#/capita)** | 63.2 (35.0) | 65.4 (45.7) | 0.00 [h] |
| **Servings Unhealthy (#/capita)** | 119.7 (74.7) | 139.8 (95.8) | 0.00 [h] |
| **Quantity-Quality Nutritional Profile**[f] | | | |
| **Protein (g/capita)** | 677.8 (338.3) | 729.3 (458.1) | 0.00 [h] |
| **Protein Healthy (g/capita)** | 337.4 (204.6) | 344.2 (259.2) | 0.076 |
| **Protein Unhealthy (g/capita)** | 340.4 (193.9) | 385.1 (255.6) | 0.00 [h] |
| **Carbohydrates (g/capita)** | 3522.3 (1974.2) | 3804.0 (2429.7) | 0.00[h] |
| **Carbohydrates Healthy(g/capita)** | 1165.4 (732.6) | 1188.2 (925.1) | 0.096 |
| **Carbohydrates Unhealthy (g/capita)** | 2356.9 (1563.1) | 2615.8 (1832.7) | 0.00 [h] |
| **Fat (g/capita)** | 832.5 (471.0) | 922.1 (608.2) | 0.00 [h] |
| **Fat Healthy (g/capita)** | 247.5 (155.3) | 256.5 (219.1) | 0.00 [h] |
| **Fat Unhealthy (g/capita)** | 585.0 (373.4) | 665.6 (452.2) | 0.00 [h] |
| **Fiber (g/capita)** | 130.4 (78.6) | 140.8 (94.3) | 0.00 [h] |
| **Fiber Healthy (g/capita)** | 47.2 (44.9) | 48.6 (48.5) | 0.093 |
| **Fiber Unhealthy (g/capita)** | 83.2 (53.0) | 92.2 (63.9) | 0.00[h] |
| **Sodium (mg/capita)** | 37526.4 (19377.2) | 42407.4 (30159.6) | 0.00 [h] |
| **Sodium Healthy (g/capita)** | 14894.7 (8456.1) | 16435.2 (15376.7) | 0.00 [h] |
| **Sodium Unhealthy (g/capita)** | 22631.7 (13406.1) | 25972.2 (17925.3) | 0.00 [h] |
| **Calories (kcal/capita)** | 24023.3 (12719.8) | 26117.3 (16227.5) | 0.00 [h] |
| **Calories Healthy (kcal/capita)** | 8211.0 (4625.3) | 8412.9 (6270.5) | 0.02 |
| **Calories Unhealthy (kcal/capita)** | 15812.3 (9821.9) | 17704.4 (11712.1) | 0.00 [h] |
| **Quality Nutritional Profiles**[g] | | | |
| **Protein (g/serving)** | 3.8 (0.84) | 3.8 (0.82) | 0.00 [h] |
| **Protein Healthy (g/serving)** | 1.91 (0.80) | 1.84 (0.76) | 0.00 [h] |
| **Protein Unhealthy (g/serving)** | 1.89 (0.55) | 1.91 (0.55) | 0.09 |
| **Carbohydrates (g/serving)** | 19.4 (3.3) | 18.7 (3.3) | 0.00 [h] |
| **Carbohydrates Healthy(g/serving)** | 6.7 (3.0) | 6.1 (2.8) | 0.00 [h] |

| | | | |
|---|---|---|---|
| **Carbohydrates Unhealthy (g/serving)** | 12.7 (3.4) | 12.6 (3.4) | 0.16 |
| **Fat (g/serving)** | 4.6 (1.0) | 4.6 (1.0) | 0.02 |
| **Fat Healthy (g/serving)** | 1.4 (0.59) | 1.3 (0.57) | 0.00 [h] |
| **Fat Unhealthy (g/serving)** | 3.2 (0.97) | 3.3 (0.96) | <0.00 [h] |
| **Fiber (g/serving)** | 0.8 (0.32) | 0.6 (0.3) | 0.00 [h] |
| **Fiber Healthy (g/serving)** | 0.3 (0.24) | 0.3 (0.22) | 0.00 [h] |
| **Fiber Unhealthy (g/serving)** | 0.5 (0.19) | 0.76 (.30) | 0.19 |
| **Sodium (mg/serving)** | 220.7 (50.0) | 212.3 (120.0) | 0.03 |
| **Sodium Healthy (mg/serving)** | 85.0 (34.0) | 84.8 (120.3) | 0.91 |
| **Sodium Unhealthy (mg/serving)** | 124.8 (36.6) | 127.5 (37.0) | 0.00 h |
| **Calories (kCal/serving)** | 132.6 (16.5) | 128.8 (17.1) | 0.00 [h] |
| **Calories Healthy (kcal/serving)** | 46.7 (16.6) | 42.7 (15.8) | 0.00 [h] |
| **Calories Unhealthy (kcal/serving)** | 85.9 (20.1) | 86.1 (19.6) | 0.18 |

kcal: Kilocalorie; g: gram, mg: milligram

[a]15 food categories include: CSDs, cereals, crackers, ice cream, milk, juices, salty snacks, soup, cheese, cookies, frozen dinners, processed meats, yogurt, baked goods/desserts, candy/confectionary

[b] Perceived Healthy= Cereal, cheese, crackers, juices, milk, soup, and yogurt

[c] Perceived Unhealthy= cookies, CSDs, ice cream, frozen dinners, processed meats, salty snacks, baked goods/desserts, candy/confectionary

[d] P-value calculated using Chi square and 2-sample T-test for categorical and continuous variables, respectively

[e] Expressed as one-year monthly averaged weighed serving per capita

[f] Expressed as one-year monthly average weighed per capita

[g] Expressed as one-year monthly average weighed per capita, per serving

[h] Statistically significant using Holms step-down correction applied for multiple comparison across 15 food and drink categories
