# Supplementary material for: Quantifying differences in packaged food and drink purchases among households with diet-related cardiometabolic multi-morbidity: a cross-sectional analysis
Source: BMC Public Health. 2022 Nov 17;22:2101. doi: 10.1186/s12889-022-14626-3 (PMC9670385; doi:10.1186/s12889-022-14626-3)
Supplement: Supplementary file 8 — Additional file 8. Regression coefficients and adjusteda odds ratios of diet-related cardiometabolic multi-morbid households relative to 100 kcal per capita, per month, estimated by various residual modelsb, for purchases from 3 calorie sources, fiber, sodium, and calories from all food & drinksc and when grouped into perceived healthyd & unhealthye when including baked goods/desserts and candy/confectionary items. Supplemental analysis re-running multiple variable logistic regression substitution models using residuals with the inclusion of baked goods/desserts and candy/confectionary food items. [file 12889_2022_14626_MOESM8_ESM.pdf]

Additional File 8. Regression coefficients and adjusted[a] odds ratios of diet-related cardiometabolic multi-morbid households relative to 100 kcal per capita, per month, estimated by various residual models[b], for purchases from 3 calorie sources, fiber, sodium, and calories from all food & drinks[c] and when grouped into perceived healthy[d] & unhealthy[e] when including baked goods/desserts and candy/confectionary items

		**Included**	**B x 100 kcal**	**Odds Ratio**	**Standard Error**	**95 % Confidence Interval**	**P Value**
		**Across all 15 Food & Drink Categories**					
	**Excluded**	Fat	0.01	1.01	0.00	1.00, 1.02	0.002[g]
**Model 1**	**Protein**	Carbohydrates	0.01	1.01	0.00	1.00, 1.02	0.000 [g]
		Fiber Residual	-0.03	0.97	0.01	0.95, 0.99	0.007 [g]
		Sodium Residual	0.11	1.12	0.01	1.01, 1.15	0.000 [g]
		Calories	0.00	1.00	0.00	1.00, 1.00	0.000 [g]
**Model 2**	**Carbohydrates**	Fat	-0.00	0.99	0.00	0.99, 1.00	0.546
		Protein	-0.01	0.99	0.00	0.98, 1.00	0.003 [g]
		Fiber Residual	-0.03	0.97	0.01	0.95, 0.99	0.011 [g]
		Sodium Residual	0.11	1.11	0.01	1.09, 1.14	0.000 [g]
		Calories	0.00	1.00	0.00	1.00, 1.00	0.000 [g]
**Model 3**	**Fat**	Protein	-0.01	0.99	0.00	0.98, 1.00	0.031
		Carbohydrates	0.00	1.00	0.00	0.99, 1.01	0.256
		Fiber Residual	-0.03	0.97	0.01	0.95, 0.99	0.006 [g]
		Sodium Residual	0.11	1.12	0.01	1.09, 1.14	0.000 [g]
		Calories	0.00	1.00	0.00	1.00, 1.00	0.000 [g]
		**Across Perceived Healthy Food & Drink Categories**					
	**Excluded**	Fat	-0.00	0.99	0.01	0.99, 1.01	0.833
**Model 1a**	**Protein**	Carbohydrates	-0.00	0.99	0.01	0.99, 1.01	0.799
		Fiber	-0.02	0.98	0.01	0.96, 1.00	0.092
		Sodium	0.08	1.08	0.01	1.06, 1.12	0.000 [g]
		Healthy Calories	0.00	1.00	0.00	1.00, 1.00	0.184
**Model 2a**	**Carbohydrates**	Total Fat	0.00	1.000	0.00	0.99, 1.01	0.992
		Protein	0.00	1.002	0.01	0.99, 1.01	0.751
		Fiber	-0.02	0.981	0.01	0.96, 1.00	0.067
		Sodium	0.08	1.085	0.01	1.06, 1.11	0.000 [g]
		Healthy Calories	0.00	1.001	0.00	1.00, 1.00	0.184
**Model 3a**	**Fat**	Protein	0.00	1.00	0.01	0.99, 1.01	0.776
		Carbohydrates	0.00	1.00	0.00	0.99, 1.01	0.986
		Fiber	-0.02	0.99	0.01	0.96, 1.00	0.080
		Sodium	0.08	1.09	0.01	1.06, 1.11	0.000 [g]
		Healthy Calories	0.00	1.00	0.00	1.00, 1.00	0.183
		**Across Perceived Unhealthy Food & Drink Categories**					
	**Excluded**	Fat	0.04	1.04	0.01	1.02, 1.06	0.000 [g]
**Model 1b**	**Protein**	Carbohydrates	0.04	1.04	0.01	1.02, 1.05	0.000 [g]
		Fiber	-0.00	0.99	0.01	0.98, 1.02	0.825
		Sodium	0.11	1.13	0.01	1.01, 1.16	0.000 [g]

		Unhealthy Calories	0.00	1.00	0.00	1.001, 1.002	0.000 [g]
**Model 2b**	**Carbohydrates**	Fat	-0.00	0.99	0.00	0.99, 1.00	0.469
		Protein	-0.04	0.97	0.01	0.95, 0.99	0.001 [g]
		Fiber	-0.01	0.99	0.00	0.97, 1.01	0.374
		Sodium	0.12	1.12	0.02	1.09, 1.16	0.000 [g]
		Unhealthy Calories	0.00	1.00	0.00	1.00, 1.00	0.000 [g]
**Model 3b**	**Fat**	Protein	-0.03	0.97	0.01	0.95, 0.99	0.004 [g]
		Carbohydrates	0.00	1.00	0.00	0.99, 1.01	0.214
		Fiber	-0.01	0.99	0.01	0.97, 1.01	0.380
		Sodium	0.12	1.12	0.02	1.09, 1.16	0.000 [g]
		Unhealthy Calories	0.00	1.00	0.00	1.00, 1.00	0.000 [g]

[a]Models adjusted for household size, head(s) of household BMI, maximum age for head(s) of household, self-identified race/ethnicity of household, maximum education attainment for head(s) of household, household income, marital status, physical activity, year of data collection, and average total calories per capita available from categories of packaged foods and drinks perceived to be healthy and unhealthy in the home each month

[b]Nutrient residuals for energy bearing nutrients computed using linear regression: kcal of each nutrient over all packaged foods and drinks purchased per month was regressed on the average total calories over all packaged foods and beverages purchased per month. Nutrient residuals are independent of total calories. Point estimates for nutrient residuals reflect a 100-kcal per capita increase. For perceived healthy and unhealthy food and drink categories, the same methods described above were used, separately for healthy and unhealthy categories

[c]Nutrient residuals for sodium and fiber computed using linear regression: grams (or milligrams) of each nutrient over all packaged foods and drinks purchased per month was regressed on the average total calories over all packaged foods and drinks purchased per month. Nutrient residuals are independent of total calories. Point estimates for nutrient residuals reflect an increase per each 0.5 standard deviation per each residual. For sodium and fiber from perceived healthy and unhealthy food and drink categories, the same methods described above were used, separately for healthy and unhealthy categories

[d]15 food categories include: CSDs, cereals, crackers, ice cream, milk, juices, salty snacks, soup, cheese, cookies, frozen dinners, processed meats, yogurt, baked goods/desserts, candy/confectionary

[e] Perceived Healthy= Cereal, cheese, crackers, juices, milk, soup, and yogurt

[f] Perceived Unhealthy= cookies, CSDs, ice cream, frozen dinners, processed meats, salty snacks, baked goods/desserts, and candy/confectionary

[g] Statistically significant using Holm’s step-down approach for multiple comparisons
