# Supplementary material for: Quantifying differences in packaged food and drink purchases among households with diet-related cardiometabolic multi-morbidity: a cross-sectional analysis
Source: BMC Public Health. 2022 Nov 17;22:2101. doi: 10.1186/s12889-022-14626-3 (PMC9670385; doi:10.1186/s12889-022-14626-3)
Supplement: Supplementary file 10 — Additional file 10. Adjusteda odds ratios of diet-related cardiometabolic multimorbid households relative to monthly weighed per capita nutrients per serving, controlling for calories per capita from all food & drinksb and when grouped into perceived healthyc and unhealthyd, excluding carbonated soft drinks and juices and including baked goods/desserts and candy/confectionary. Supplemental analysis re-running multiple variable logistic regression model using per serving (quality) variables without carbonated soft drink and juices but with the inclusion of baked goods/desserts and candy/confectionary food items. [file 12889_2022_14626_MOESM10_ESM.pdf]

Additional file 10. Adjusted[a] odds ratios of diet-related cardiometabolic multimorbid households relative to monthly weighed per capita nutrients per serving, controlling for calories per capita from all food & drinks[b] and when grouped into perceived healthy[c] and unhealthy[d] , excluding carbonated soft drinks and juices and including baked goods/desserts and candy/confectionary

	Coefficient	Odds Ratio	Standard Error	95% Confidence Interval	P Value[e]
**Model 1: Across all 13 Food & Drink Categories**					
**Protein (g/serving)**	-0.11	0.90	0.02	0.85, 0.95	0.000 [e]
**Carbohydrates (g/serving)**	-0.04	0.96	0.01	0.95, 0.98	0.000 [e]
**Fat (g/serving)**	-0.03	0.97	0.02	0.94, 1.00	0.091
**Fiber (g/serving)**	-0.02	0.98	0.07	0.85, 1.11	0.794
**Sodium (mg/serving)**	0.00	1.00	0.00	1.00, 1.00	0.000 [e]
**Calories (kcal/capita- 0.5 SD Kcal)**	0.05	1.05	0.01	1.03, 1.07	0.000 [e]
**Model 2: Perceived Healthy Food & Drinks**					
**Protein (g/serving)**	-0.08	0.92	0.03	0.87, 0.97	0.001 [e]
**Carbohydrates (g/serving)**	-0.01	0.99	0.01	0.97, 1.01	0.150
**Fat (g/serving)**	-0.06	0.95	0.03	0.89, 0.99	0.036
**Fiber (g/serving)**	-0.20	0.82	0.08	0.66, 0.98	0.015 [e]
**Sodium (mg/serving)**	0.00	1.00	0.00	1.00, 1.00	0.000 [e]
**Calories Healthy (kcal/capita- 0.5 SD Kcal)**	0.00	1.00	0.00	1.00, 1.00	0.000 [e]
**Model 3: Perceived Unhealthy Food & Drinks**					
**Protein (g/serving)**	-0.11	0.89	0.05	0.80, 0.99	0.021
**Carbohydrates (g/serving)**	-0.04	0.96	0.01	0.94, 0.98	0.000 [e]
**Fat (g/serving)**	0.03	1.03	0.02	0.98, 1.08	0.197
**Fiber (g/serving)**	0.26	1.30	0.16	0.98, 1.62	0.105
**Sodium (mg/serving)**	0.00	1.00	0.00	1.00, 1.00	0.000 [e]
**Calories Unhealthy (kcal/capita- 0.5 SD Kcal)**	0.00	1.00	0.00	1.00, 1.00	0.000 [e]

kcal: Kilocalorie; SD: Standard deviation.

[a] Models adjusted for household size, head(s) of household BMI, maximum age for head(s) of household, self-identified race/ethnicity of household, maximum education attainment for head(s)of household, household income, marital status, physical activity, year of data collection, and average total calories per capita available from packaged food and drinks in the home each month

[b]13 food categories include: Cereals, crackers, ice cream, milk, salty snacks, soup, cheese, cookies, frozen dinners, processed meats, yogurt, baked goods/desserts, candy/confectionary

[c] Perceived Healthy= Cereal, cheese, crackers, milk, soup, and yogurt

[d] Perceived Unhealthy= cookies, ice cream, frozen dinners, processed meats, salty snacks, baked goods/desserts, candy/confectionary

[e] Statistically significant using Holms step-down correction applied for multiple comparison across 13 food and drink categories
